# Novel p.G250A Mutation Associated with Chronic Pancreatitis Highlights Misfolding-Prone Region in Carboxypeptidase A1 (CPA1)

**DOI:** 10.3390/ijms232415463

**Published:** 2022-12-07

**Authors:** Máté Sándor, Franziska G. Thiel, Margit Schmid, Alexandra Demcsák, Nataly C. Morales Granda, Balázs Csaba Németh, Sandor Vajda, André Hoerning, Miklós Sahin-Tóth

**Affiliations:** 1 Department of Surgery, University of California Los Angeles, Los Angeles, CA 90095, USA; 2 Department of Medicine, University Medicine Greifswald, 17475 Greifswald, Germany; 3 Clinic for Children and Adolescent Medicine, Friedrich Alexander University Erlangen-Nuremberg, 91054 Erlangen, Germany; 4 Department of Biomedical Engineering, Boston University, Boston, MA 02215, USA

**Keywords:** pancreatitis, misfolding, endoplasmic reticulum stress, carboxypeptidase, genetic risk

## Abstract

Inborn mutations in the digestive protease carboxypeptidase A1 (CPA1) gene may be associated with hereditary and idiopathic chronic pancreatitis (CP). Pathogenic mutations, such as p.N256K, cause intracellular retention and reduced secretion of CPA1, accompanied by endoplasmic reticulum (ER) stress, suggesting that mutation-induced misfolding underlies the phenotype. Here, we report the novel p.G250A *CPA1* mutation found in a young patient with CP. Functional properties of the p.G250A mutation were identical to those of the p.N256K mutation, confirming its pathogenic nature. We noted that both mutations are in a catalytically important loop of CPA1 that is stabilized by the Cys248-Cys271 disulfide bond. Mutation of either or both Cys residues to Ala resulted in misfolding, as judged by the loss of CPA1 secretion and intracellular retention. We re-analyzed seven previously reported CPA1 mutations that affect this loop and found that all exhibited reduced secretion and caused ER stress of varying degrees. The magnitude of ER stress was proportional to the secretion defect. Replacing the naturally occurring mutations with Ala (e.g., p.V251A for p.V251M) restored secretion, with the notable exception of p.N256A. We conclude that the disulfide-stabilized loop of CPA1 is prone to mutation-induced misfolding, in most cases due to the disruptive nature of the newly introduced side chain. We propose that disease-causing *CPA1* mutations exhibit abolished or markedly reduced secretion with pronounced ER stress, whereas *CPA1* mutations with milder misfolding phenotypes may be associated with lower disease risk or may not be pathogenic at all.

## 1. Introduction

Digestive carboxypeptidases are zinc-dependent metalloproteases that remove C-terminal amino acids from dietary protein and peptide substrates [1,2,3,4,5]. Digestion of proteins with trypsin exposes C-terminal Arg and Lys residues, which are then cleaved off by carboxypeptidase B1 (CPB1). Digestion with chymotrypsin and elastase generates C-terminal aromatic and aliphatic amino-acids that are further hydrolyzed by carboxypeptidase A1 (CPA1) and A2 (CPA2), which exhibit overlapping specificity. Carboxypeptidases are secreted as inactive proenzymes and together with trypsinogens represent the most abundant digestive zymogens in the pancreatic juice [6]. Physiological activation in the duodenum is initiated by trypsin [2,3,4]; however, full activation of proCPA1 and proCPA2, but not of proCPB1, requires further proteolysis of the activation peptide by chymotrypsin C (CTRC) [7]. Human proCPA1 and proCPA2, but not proCPB1, form complexes with chymotrypsin-like proelastase 3B (proCELA3B) [1,8,9,10]. Interestingly, proCELA3A, which shares 92% identity with proCELA3B, does not form stable complexes due to the evolutionary replacement of Ala241 with Gly [10]. Since position 241 is polymorphic in both proelastases, individual variations in complex formation are likely [11]. Complex formation further stabilizes the activation peptide of proCPA1 and proCPA2 and thereby protects against premature activation [10]. In ruminants, stable ternary complexes consisting of proCPA1, proCELA3B, and the CTRC precursor have been described [4,12].

In 2013, sequence analysis of the *CPA1* gene in patients with chronic pancreatitis (CP) demonstrated an association between loss-of-function variants and early-onset disease [13]. The large effect size (odds ratio ~25 for the entire cohort and 84 for the subgroup under the age 10) indicated that *CPA1* variants were strong genetic risk factors. Indeed, subsequent studies confirmed that *CPA1* variants could cause autosomal dominant hereditary pancreatitis [14,15]. No association could be demonstrated between *CPA2* or *CPB1* variants and CP [16], although *CPB1* and some *CPA1* variants may be associated with pancreatic cancer [17,18]. Functional analysis of *CPA1* variants revealed that most mutations caused reduced proCPA1 secretion, and studies on the most frequently found variant, p.N256K, identified misfolding-induced endoplasmic reticulum (ER) stress as a potential disease mechanism [13]. Diminished secretion, intracellular retention, and cellular ER stress were subsequently demonstrated for *CPA1* variants p.S282P [14] and p.K374E [15], associated with hereditary pancreatitis. A mouse model carrying the p.N256K mutation in the mouse *Cpa1* gene developed spontaneous, progressive CP with moderate acinar atrophy, macrophage infiltration, diffuse fibrosis, acinar-to-ductal metaplasia, serum amylase elevations, and mild ER stress [19]. Taken the cell culture and mouse studies together, available evidence indicates that misfolding *CPA1* variants cause CP through chronic ER stress, as exemplified by the p.N256K variant.

Since the initial discovery, there has been an increasing number of *CPA1* variants reported, and determination of potential pathogenicity has been a challenge to researchers and clinicians alike [13,14,15,20,21]. Due to the low frequency of most *CPA1* variants, genetic studies alone cannot prove or rule out disease association. Therefore, functional analysis is required to assess clinical significance. Using the p.N256K variant as the pathogenic reference mutation, one can compare functional properties of new *CPA1* variants and classify variants as pathogenic if their behavior mimics p.N256K. However, a quantitative relationship between the biological impact of *CPA1* variants and their cell culture effects has not been established. Here, we report the functional characterization of a novel *CPA1* mutation found in a case of early-onset CP. Furthermore, we identify the disulfide-stabilized surface loop of CPA1 as a misfolding-prone region, frequently targeted by natural mutations. Finally, we propose that true pathogenic *CPA1* mutations tend to cause severe secretion defects and strong ER stress in cell culture experiments, while variants with lesser effects may not be pathogenic at all.

## 2. Results

### 2.1. A Novel CPA1 Mutation in an Early-Onset Case of CP

The female index patient was diagnosed with recurrent acute pancreatitis episodes in 2020, at the age of 17, based on characteristic abdominal pain triggered by eating, and elevated serum lipase levels. Repeated abdominal imaging (ultrasound, magnetic resonance cholangiopancreatography, and endoscopic ultrasound) showed no pathological changes in the pancreas parenchyma, the Wirsung duct, or the bile ducts. Sweat chloride concentration was normal. Pancreatic elastase levels in stool were in the normal range. Celiac and viral serology were negative. The patient’s parents had no history of pancreas disease, however, the maternal grandfather died of pancreatic carcinoma. Genetic testing revealed no pathogenic variants in the *PRSS1*, *SPINK1*, *CTRC*, *CFTR*, *CLDN2*, *CASR*, and *LPL* genes. In the *CPA1* gene, the novel heterozygous c.749G>C (p.G250A) variant was identified. The parents were unavailable for genetic testing.

### 2.2. Functional Analysis of the p.G250A Mutation

**Figure 1:**
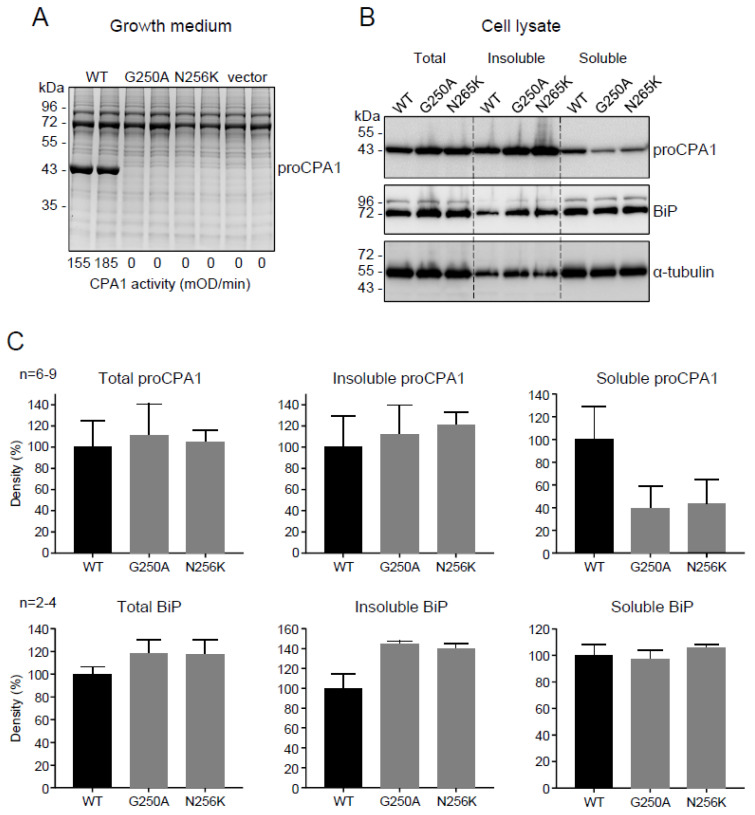
Effect of the p.G250A *CPA1* variant on cellular secretion, enzyme activity, intracellular protein levels, solubility, and BiP protein levels in transfected HEK 293T cells. For comparison, wild-type CPA1 (WT), the reference pathogenic mutation p.N256K, and empty expression vector were studied. (**A**) Secretion of proCPA1 to the growth medium analyzed by SDS-PAGE and Coomassie Blue staining. CPA1 enzyme activity in the medium after activation of proCPA1 is indicated in milliOD/min units. (**B**) Protein levels of proCPA1, BiP, and α-tubulin in total cell lysates, and in the insoluble and soluble cellular fractions. Proteins were detected by Western blotting. (**C**) Densitometric evaluation of proCPA1 and BiP levels in total cell lysates, and in the insoluble and soluble cellular fractions.

**Figure 2:**
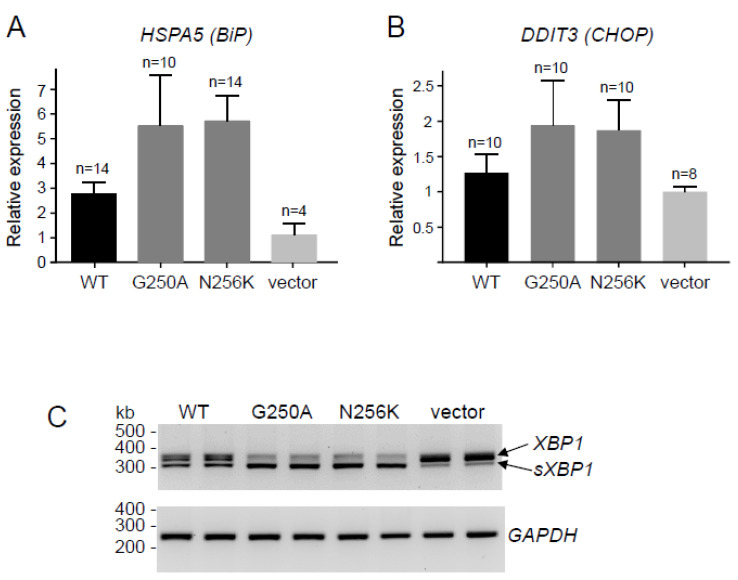
Effect of the p.G250A *CPA1* variant on endoplasmic reticulum stress markers in transfected HEK 293T cells. For comparison, the effect of wild-type CPA1 (WT), the reference pathogenic mutation p.N256K, and empty expression vector were studied. (**A**) Expression of *HSPA5 (BiP)* mRNA measured by RT-qPCR. (**B**) Expression of *DDIT3 (CHOP)* mRNA measured by RT-qPCR. (**C**) Splicing of *XBP1* mRNA (sXBP1) assessed by RT-PCR and agarose gel electrophoresis. *GAPDH* mRNA was measured as a loading control. Representative gel from three experiments is shown.

To evaluate the effects of the p.G250A mutation on CPA1 expression and function, we transiently transfected HEK 293T cells and measured the levels of secreted proCPA1 in the conditioned medium using SDS-PAGE and enzymatic activity assays. We used the known pathogenic mutant p.N256K as positive reference control, and cells transfected with empty vector served as negative control. We found that gels stained with Coomassie Blue showed strong bands for wild-type proCPA1 whereas no secreted protein was detected in the medium of cells transfected with p.N256K, p.G250A, or empty vector (Figure 1A). CPA1 activity of the conditioned medium was high after activation when cells were transfected with wild-type CPA1, but no activity was detectable in the medium of cells expressing the p.G250A and p.N256K mutants or containing empty vector (Figure 1A). Western blot analyses of cell lysates revealed that expression levels of wild-type proCPA1 were comparable to those of mutants p.G250A and p.N256K (Figure 1B,C). Separation of the cell lysates into soluble and insoluble fractions by centrifugation demonstrated that the p.G250A and p.N256K mutants were less soluble and had a higher tendency to form insoluble aggregates relative to wild-type proCPA1 (Figure 1B,C). The lack of secretion, intracellular retention, and higher propensity for aggregation suggested that CPA1 mutants p.G250A and p.N256K suffered misfolding. Consistent with this interpretation, cells expressing these mutants showed higher levels of the ER master chaperone BiP and contained more BiP in the insoluble fraction relative to cells transfected with wild-type CPA1 or vector only (Figure 1B,C). In agreement with the results of the Western blot analyses of the BiP protein, RT-qPCR also demonstrated that the *CPA1* mutations induced considerable elevations in the mRNA levels for *HSPA5* (*BiP*) (Figure 2A), and the ER-stress associated pro-apoptotic transcription factor *DDIT3* (*CHOP*) (Figure 2B). Splicing of the transcription factor *XBP1* mRNA was also markedly increased in cells transfected with mutant CPA1 constructs versus wild-type CPA1 or vector only (Figure 2C). Taken together, the observations indicate that the *CPA1* mutation p.G250A causes enzyme misfolding and ER stress to the same extent as the known pathogenic mutation p.N256K. Thus, considering the clinical picture, mutation p.G250A should be classified as a pathogenic variant with respect to CP.

### 2.3. The Disulfide-Stabilized Loop in CPA1 Is Essential for Folding

**Figure 3:**
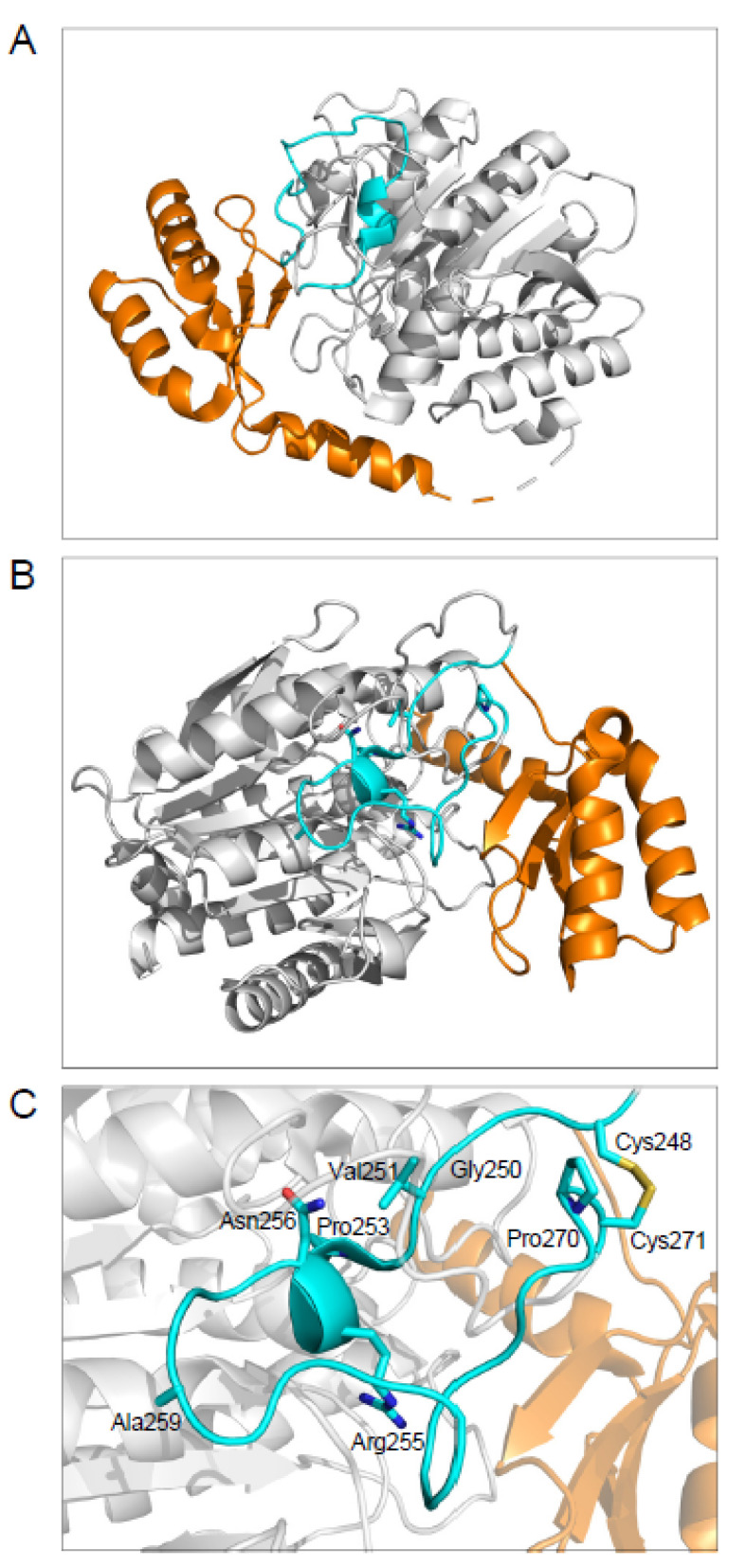
Structure of human pancreatic proCPA1. (**A**) Side view (Protein Data Bank code 5OM9). The propeptide (residues 17–110) is highlighted in orange, the loop formed by residues 248-271 is in cyan, and the rest of the structure is shown as grey cartoon. (**B**) Top view with emphasis on the loop stabilized by the disulfide bridge between residues Cys248 and Cys271. (**C**) Magnified view of the 248-271 loop. The side chains shown in stick representation are Cys248, Val251, Arg255, Asn256, Ala259, Pro270, and Cys271. The position of Gly250 is also indicated.

**Figure 4:**
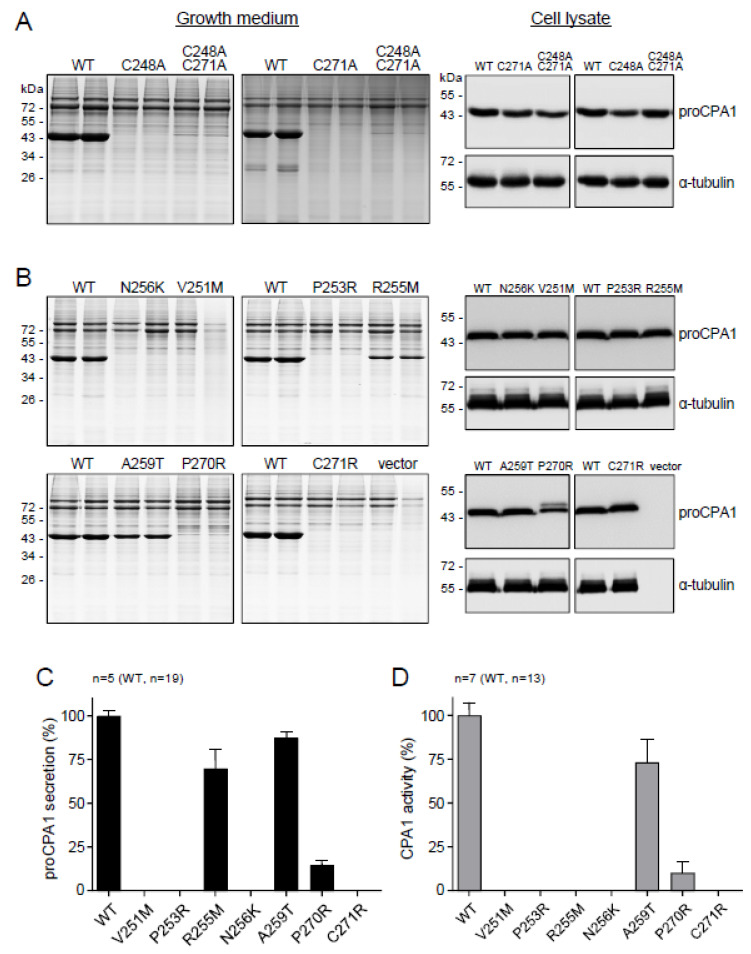
Effect of *CPA1* variants on cellular secretion, intracellular protein levels, and enzyme activity. Secretion of proCPA1 protein from transfected HEK 293T cells was assessed by SDS-PAGE and Coomassie Blue staining. Protein levels of proCPA1 in cell lysates were analyzed by Western blotting. Alpha-tubulin was measured as loading control. (**A**) Effect of single Cys-mutations C248A and C271A, and double mutation C248A, C271A. (**B**) Effect of natural *CPA1* variants p.V251M, p.P253R, p.R255M, p.N256K, p.A259T, p.P270R, and p.C271R. (**C**) Densitometric analysis of proCPA1 secretion to the growth medium. (**D**) CPA1 enzyme activity in the conditioned medium after activation of proCPA1. WT, wild-type.

We noted that both the novel mutation p.G250A and the reference pathogenic mutation p.N256K are located within a loop segment that is stabilized by the sole Cys248-Cys271 disulfide bond in CPA1 (Figure 3). To examine the significance of this disulfide bridge in CPA1 folding, we mutated the Cys residues individually or in combination to Ala. Strikingly, proCPA1 secretion from transfected cells was abolished by the single mutations p.C248A and p.C271A, or the double mutation p.C248A, C271A (Figure 4A). All Cys mutants were detected at normal levels in cell lysates, indicating that the secretion defect was due to misfolding and consequent intracellular retention.

### 2.4. CPA1 Mutations within the Disulfide-Stabilized Loop

We surveyed the literature for natural mutations identified in this region. We found that besides p.G250A and p.N256K, six other CPA1 mutations have been reported within the Cys248-Cys271 sequence [13]. Mutations p.V251M, p.P253R, p.R255M, p.P270R, and p.C271R were detected in patients with CP, whereas mutation p.A259T was found in a subject without pancreatic disease (Table 1). No *CPA1* mutation was found in this region in association with pancreatic cancer. After the completion of our experiments, we noted that mutation p.A259V was also described in a healthy control [22]. We did not include this mutation in our studies, but it likely has similar properties as the p.A259T mutation. The position of the *CPA1* mutations in the protein structure is shown in Figure 3. Mutations p.V251M, p.P253R, p.R255M, p.A259T, p.P270R, and p.C271R were previously characterized with respect to cellular secretion and enzyme activity (Table 2) [13]; however, intracellular levels and their propensity to induce ER stress were not reported. Therefore, we re-examined the effects of these six mutations together with the p.N256K pathogenic reference mutation. In the conditioned medium of transfected cells, no proCPA1 secretion was detected for mutants p.V251M, p.P253R, p.N256K, and p.C271R, whereas secretion of mutant p.P270R was detectable but markedly reduced (14%) relative to wild-type CPA1. In contrast, mutants p.R255M and p.A259T were much better secreted to the conditioned medium; reaching 70% and 87% of wild-type proCPA1 levels, respectively (Figure 4B,C, Table 2). All mutants were readily detectable in cell lysates by Western blotting, at levels that were comparable to those of wild-type CPA1 (Figure 4B). Interestingly, mutant p.P270R migrated as a doublet on gels due to partial glycosylation (see Discussion). As predicted by the absence of the proCPA1 protein, no appreciable enzyme activity was measurable in the activated conditioned medium of mutants p.V251M, p.P253R, p.N256K, and p.C271R, and the medium with mutant p.P270R exhibited greatly reduced activity (10% of wild-type CPA1) (Figure 4D, Table 2). Considering the two secreted mutants, only mutant p.A259T showed high CPA1 activity (73% of wild-type), whereas mutant p.R255M was catalytically inactive (Figure 4D, Table 2).

**Figure 5:**
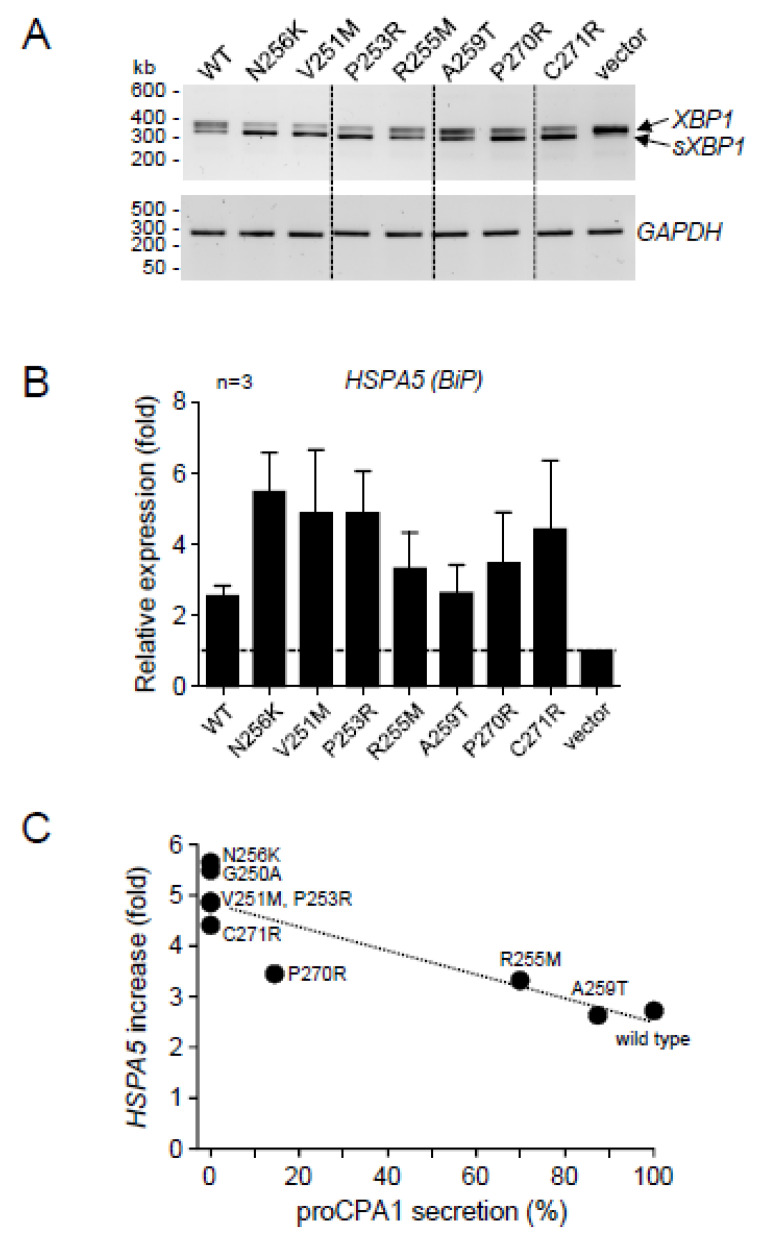
Effect of natural *CPA1* variants on endoplasmic reticulum stress markers in transfected HEK 293T cells. (**A**) Splicing of *XBP1* mRNA (*sXBP1*) assessed by RT-PCR and agarose gel electrophoresis. *GAPDH* mRNA was measured as a loading control. Representative gel from two experiments is shown. (**B**) Expression of *HSPA5 (BiP)* mRNA measured by RT-qPCR. (**C**) Relationship between proCPA1 secretion and *HSPA5 (BiP)* mRNA levels. WT, wild-type.

To evaluate the ER stress caused by the expression of the *CPA1* mutants, we assessed splicing of *XBP1* mRNA by RT-PCR and agarose gel electrophoresis (Figure 5A), and measured *HSPA5* (*BiP*) mRNA levels by RT-qPCR (Figure 5B). Relative to cells transfected with empty vector only, wild-type and mutant CPA1 constructs induced varying levels of ER stress, which seemed inversely proportional to the amount of CPA1 secreted, i.e., directly proportional with the extent of the secretion defect. This was best visualized when the increase in *HSPA5* (*BiP*) expression (fold change relative to cells transfected with vector only) was plotted as a function of CPA1 secretion (% of wild-type CPA1). In this analysis, the presumably pathogenic mutations, including p.G250A, clustered around the pathogenic reference mutation p.N256K in the upper left corner of the graph, characterized by the complete absence of secretion and around 5-fold increase in *HSPA5* (*BiP*) expression (Figure 5C).

### 2.5. Ala Replacements Rescue Most CPA1 Mutants

**Figure 6:**
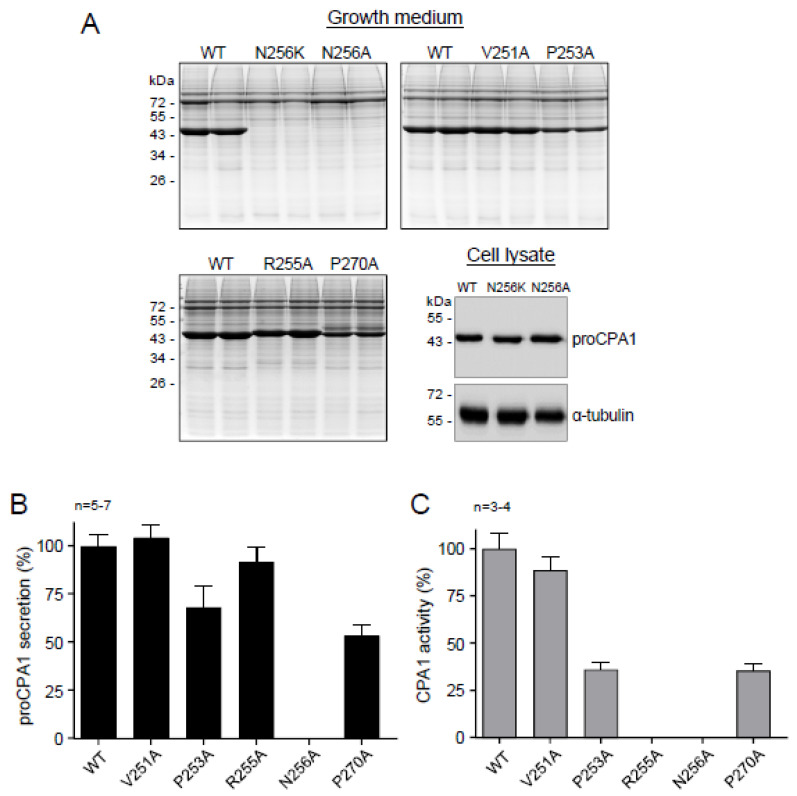
Effect of *CPA1* Ala-variants on cellular secretion and enzyme activity. Secretion of proCPA1 protein from transfected HEK 293T cells was assessed by SDS-PAGE and Coomassie Blue staining. Protein levels of the p.N256A variant in cell lysates were analyzed by Western blotting. Alpha-tubulin was measured as a loading control. (**A**) Effect of *CPA1* variants p.V251A, p.P253A, p.R255A, p.N256A, and p.P270A. For comparison, variant p.N256K was also included. (**B**) Densitometric analysis of proCPA1 secretion to the growth medium. (**C**) CPA1 enzyme activity in the conditioned medium after activation of proCPA1. WT, wild-type.

The experiments described so far established that the loop stabilized by the Cys248-Cys271 disulfide bridge is required for proper CPA1 folding, and mutations in this region often cause misfolding, reduced secretion, and ER stress. To clarify why mutations tend to be disruptive here, we introduced Ala replacements at the positions where the natural mutations were found. If the native side chain was essential for folding, an Ala mutation would be just as detrimental as the natural mutation. Conversely, if extra bulk and/or charge introduced by the natural mutations were disruptive, then replacement with the small and neutral Ala would be expected to rescue the misfolding phenotype and restore secretion. In these experiments, positions 251, 253, 255, 256, and 270 were analyzed, where natural mutations introduced Arg, Lys, or Met residues. With the notable exception of p.N256A, Ala mutants showed greatly improved secretion relative to the respective natural mutants (Figure 6A,B). Mutant p.N256A was not secreted but was readily detected in cell lysates (Figure 6A,B). Enzyme activity assays of the activated conditioned medium revealed that mutant p.V251A was as active as wild-type CPA1, however, mutants p.P253A and p.P270A exhibited reduced CPA1 activity relative to their protein levels. Mutant p.R255A was completely inactive, in agreement with the known catalytic role of Arg255. The observations indicate that natural mutations within the Cys248-Cys271 loop tend to cause misfolding due to the disruptive nature of the newly introduced bulky side chain.

## 3. Discussion

In this study, we report identification and functional characterization of the novel p.G250A *CPA1* mutation that was found in a case of early-onset CP. To assess the pathogenic potential of the new mutation, we compared its properties to those of the known pathogenic mutation p.N256K in cell culture experiments. The results clearly indicate that both mutations p.G250A and p.N256K exhibited the so-called misfolding phenotype [23], which involved essentially undetectable secretion, intracellular retention, a tendency for aggregation, and the ability to induce ER stress as judged by upregulation of *HSPA5 (BiP)* mRNA and BiP protein, increased *XBP1* mRNA splicing, and elevated *DDIT3 (CHOP)* mRNA expression. These characteristics are highly similar, if not identical, to those reported for *CPA1* mutations p.S282P [14] and p.K374E [15], chymotrypsin C (*CTRC*) variants p.G61R and p.A73T [24,25], human cationic trypsinogen (*PRSS1*) mutants p.L104P, p.R116C, p.S127C, and p.C139S [26,27,28], a handful of pancreatic lipase (*PNLIP*) variants including p.T221M [29,30], mutations in carboxyl ester lipase (*CEL*), and a hybrid allele between *CEL* and its adjacent pseudogene (*CEL-HYB1*) [31,32,33].

We noted that both mutations p.G250A and p.N256K were in a surface loop stabilized by the single Cys248-Cys271 disulfide bridge in CPA1 (see Figure 3). This loop also contains catalytically important residues such as Asn254 and Arg255 (Asn144 and Arg145 in legacy numbering). Mutation of either or both Cys residues to Ala resulted in loss of proCPA1 secretion with normal intracellular proCPA1 levels, indicating that the disulfide bridge is essential for proper folding. Besides p.G250A and p.N256K, we re-analyzed six previously reported *CPA1* mutations within this region with respect to cellular secretion and enzyme activity and extended their characterization to include measurements of intracellular CPA1 levels and ability to induce ER stress. We found that all *CPA1* mutations caused reduced secretion and ER stress, and the magnitude of ER stress was proportional to the secretion defect. This inverse relationship between secretion and ER stress has been observed before with *CTRC, CPA1,* and *CPB1* variants [18,25]. Importantly, when the extent of *HSPA5* (*BiP*) mRNA upregulation was plotted as a function of proCPA1 secretion, mutations with high *HSPA5* (*BiP*) levels and diminished secretion grouped together with p.N256K, suggesting that these variants are all pathogenic. Indeed, *CPA1* variants p.G250A, p.V251M, p.P253R, and p.C271R were identified in CP cases. In contrast, variants p.R255M and p.A259T were clearly separated from the pathogenic group on the graph. Properties of p.A259T, which was found in a healthy control subject, were not too different from those of wild-type CPA1, and we can conclude that it represents an innocuous, benign variant. Variant p.R255M was found in a subject with CP, and it exhibited moderately reduced secretion with measurable ER stress. However, we propose that this degree of functional defect may not be sufficient to increase CP risk, and the association with CP may have been accidental. Finally, mutation p.P270R, which was found in a CP case, presents an interesting conundrum. The mutation changes the Asn-Pro-Cys sequence to Asn-Arg-Cys, and thereby creates a new *N*-linked glycosylation site, with a non-canonical Cys residue replacing Ser/Thr within the Asn-Xaa-Ser/Thr motif. This would disrupt the Cys248-Cys271 disulfide bridge; however, the glycosylation may allow for secretion of the misfolded mutant, thereby mitigating ER stress. Indeed, the glycosylated form of the p.P270R variant was visible both on the SDS-PAGE and the Western blot of cell lysate, as bands that migrated higher (slower) than the unmodified proCPA1 band. There was low but measurable secretion, and the extent of *HSPA5* (*BiP*) upregulation was somewhat lower than seen with the pathogenic mutants, raising the possibility that p.P270R might be a variant conferring lesser disease risk or no risk at all. A caveat to this conclusion is that human pancreatic acinar cells may glycosylate the mutant differently than HEK 293T cells. Taken together, the observations suggest a quantitative relationship between pathogenicity of *CPA1* variants and their behavior in cell culture experiments with respect to secretion defect and ER stress.

Our results identified the surface loop segment between Cys248 and Cys271 as a misfolding prone region in proCPA1, which is often targeted by CP-associated natural mutations. We were curious whether these mutations caused misfolding due to the loss of an essential side chain or because of the introduction of a new side chain with larger or bulk and/or different charge. To address this question, we performed Ala replacements at positions where the natural variants created Arg, Lys, or Met residues. We found Ala substitutions rescued the secretion defect caused by the natural mutations, with the sole exception of p.N256A. Inspection of the human proCPA1 crystal structure reveals that the side chain of Asn256 forms four hydrogen bonds with nearby backbone atoms [34]. The amide nitrogen interacts with the backbone oxygen atoms of both His276 and Phe279 (N-O distances 2.9 Å and 3.1 Å, respectively), and the amide oxygen forms hydrogen bonds with the backbone NH groups of Glu283 and Ser282 (N-O distances 3.0 Å and 3.2 Å, respectively). Thus, the amide group of Asn256 is in a favorable environment defined by backbone atoms, explaining the impact of mutations on the folding of the protein. Notwithstanding the unique properties of position 256, we conclude that in most cases mutations interfere with CPA1 folding owing to the introduction of a disruptive amino-acid side chain rather than the elimination of a structurally essential residue.

The disulfide-stabilized loop in CPA1 harbors amino acids that play important catalytic roles. Thus, Asn254 and Arg255 (together with Tyr358) form the S1′ substrate binding subsite and are involved in the anchoring and neutralization of the C-terminal carboxylate of the bound substrate. Accordingly, we found that replacement of Arg255 with Met (p.R255M) or Ala (p.R255A) caused complete loss of enzymatic function. Interestingly, Ala mutants of Pro residues (p.P253A and p.P270A) showed partly reduced activity relative to their secreted protein levels. Since these positions do not directly participate in substrate binding or catalysis, the lower activity is likely due to indirect effects on folding that might alter the correct positioning of the S1′ subsite-forming residues.

The *CPA1 N256K* mouse model carrying the human p.N256K mutation in the mouse *Cpa1* gene provided direct evidence that misfolding CPA1 mutants cause pancreatic injury and inflammation [19]. The pancreatitis phenotype of the mice corresponded to slowly-progressing, relatively mild CP, with acinar cell atrophy, macrophage infiltration, acinar-to-ductal metaplasia, diffuse fibrosis, and elevated plasma amylase activity in the early phase of the disease. Unlike in the human condition, spontaneous acute pancreatitis has not been observed. A highly similar CP phenotype was reported for humanized mice harboring the carboxyl ester lipase (CEL) hybrid allele *CEL-HYB1* [35]. In *CPA1 N256K* mice, severity of CP was increased and progression was accelerated by an ethanol-containing diet, indicating that alcohol and misfolding may act on the same target [36]. Although ER stress has been suggested as the underlying disease mechanism for misfolding digestive enzyme mutants associated with CP, data from the *CPA1 N256K* model offered limited proof for this contention. Thus, *Hspa5* (*BiP*) mRNA in the pancreas was only modestly elevated throughout the disease course [19]. A more significant upregulation of *Ddit3* (*Chop*) mRNA was observed; however, more recent work indicated that global deletion of *Ddit3* (*Chop*) had no impact on the severity or progression of CP in *CPA1 N256K* mice [37]. These observations raise the possibility that ER stress, and *Ddit3* (*Chop*) upregulation in particular, may be markers rather than drivers of the disease. Our results argue against this notion, as the propensity of CPA1 mutants to induce ER stress in cell culture seem to correlate directly with their pathogenic potential.

In summary, we described a novel pathogenic *CPA1* mutation in a case of early-onset CP and identified a disulfide-stabilized loop in CPA1 that is essential for proper folding and catalytic activity. This misfolding-prone region is frequently targeted by natural mutations associated with CP.

## 4. Methods

### 4.1. Accession Numbers and Nomenclature

NM_001868.4, *Homo sapiens* carboxypeptidase A1 (CPA1), mRNA, NCBI Reference Sequence. Nucleotide numbering starts from the first nucleotide of the translation initiator ATG codon. Amino-acid numbering starts from the initiator methionine of the primary translation product. We indicate the proenzyme form of CPA1 as proCPA1.

### 4.2. Genetic Testing

The novel c.749G>C (p.G250A) *CPA1* variant was identified with next generation sequencing analysis using a molecular genetic panel of known risk genes for pancreatitis including *CASR*, *CFTR*, *CLDN2*, *CPA1*, *CTRC*, *LPL*, *PRSS1*, and *SPINK1*. Subsequently, the variant was confirmed with conventional Sanger sequencing.

### 4.3. Structural Modeling

The three-dimensional structure of human pancreatic proCPA1 was modeled based on the X-ray structure of the protein co-crystallized with a mechanism-based inhibitor (Protein Data Bank code 5OM9, resolution 1.80 Å) [34]. The inhibitor and water molecules were removed, and the structure was rendered in cartoon representation using the PyMOL molecular graphics system (Version 2.0, Schrödinger, LLC, New York, NY, USA).

### 4.4. Plasmid Construction and Mutagenesis

The expression plasmid carrying the coding DNA of human proCPA1 in the pcDNA3.1(-) vector was constructed previously [7]. Mutations were generated by overlap extension PCR mutagenesis and cloned into the expression plasmids using XhoI and BamHI restriction sites.

### 4.5. Cell Culture and Transfection

HEK 293T cells (GenHunter, Nashville, TN, USA) were cultured in 6-well tissue culture plates (1.5 × 10^6^ cells per well), in Dulbecco’s Modified Eagle Medium (DMEM) (Life Technologies, Carlsbad, CA, USA) supplemented with 10% fetal bovine serum, 4 mM glutamine and 1% penicillin/streptomycin at 37 °C. Transfections were carried out using 4 µg plasmid DNA and 10 µL Lipofectamine 2000 (Life Technologies) in 2 mL DMEM medium. Cells were incubated overnight with the transfection mix, which was replaced with 1.5 mL OptiMEM reduced serum medium (Life Technologies, Carlsbad, CA, USA) the next morning. Conditioned medium and cell lysates were harvested for analysis 48 h later.

### 4.6. Measurement of Procarboxypeptidase Secretion

Secreted proCPA1 levels in the conditioned medium were determined by sodium dodecyl sulfate (SDS) polyacrylamide gel electrophoresis (PAGE), and densitometry. Aliquots (175 μL) of the medium were precipitated with 10% trichloroacetic acid (final concentration). The protein was collected by centrifugation and dissolved in 25 μL Laemmli sample buffer containing 100 mM dithiothreitol and 150 mM NaOH. The samples were heat-denatured at 95 °C for 5 min, electrophoresed on 12% SDS-polyacrylamide gels, and stained with Brilliant Blue R-250 (Coomassie Blue). Gels were digitized on a ChemiDoc Touch Imaging System (Bio-Rad, Hercules, CA, USA) as tif files, and densitometric quantitation of proCPA1 bands was performed with the ImageJ software (version 1.53e).

### 4.7. Measurement of Carboxypeptidase Activity

Secreted proCPA1 in the conditioned medium was activated to CPA1, and the enzyme activity was measured using the N-[4-methoxyphenylazoformyl]-L-phenylalanine substrate (Bachem Americas, Torrance, CA, USA). The activation reaction was carried out with 100 nM human cationic trypsin and 50 nM human CTRC (final concentrations) for 1 h at 37 °C. The 20 μL activation mixture contained 10 µL conditioned medium, 0.1 M Tris-HCl (pH 8.0), 1 mM CaCl_2_, and 0.05% Tween 20 (final concentrations). Enzyme activity was measured as described previously [13,16], with minor modifications. Briefly, the 20 μL activation mix was supplemented with 150 μL assay buffer (0.1 M Tris-HCl (pH 8.0), 1 mM CaCl_2_, and 0.05% Tween 20) and the reaction was started by adding 30 μL of 600 µM substrate solution (90 µM final concentration). The decrease in absorbance at 350 nm was monitored for 2 min in a microplate reader and the rate of substrate cleavage was determined from the linear portion of the curve.

### 4.8. Preparation of Cell Lysates and Fractionation

After removal of the conditioned medium, cells were rinsed with 1 mL phosphate-buffered saline, suspended from the wells in 1 mL phosphate-buffered saline, and centrifuged at 850× *g* for 10 min at 4 °C. The cell pellets were resuspended in 200 µL ice-cold reporter lysis buffer (E397A, Promega, Madison, WI, 5X factory stock freshly diluted to 1X with phosphate buffered saline) supplemented with protease inhibitors (Halt Protease and Phosphatase Inhibitor Cocktail, 78444, Thermo Scientific, 100X factory stock diluted to 1X). The samples were frozen at −20 °C for 1 h, thawed on ice, 100 µL was saved as the total lysate, and 100 µL was separated into soluble and insoluble fractions by centrifuging for 10 min at 850× *g* at 4 °C. The soluble supernatant was saved, and the insoluble pellet was resuspended in 100 µL lysis buffer.

### 4.9. Western Blotting

Protein levels of proCPA1 and immunoglobulin binding protein (BiP) were evaluated by Western blotting in total, soluble, and insoluble fractions of cell lysates. Ten µL of each fraction was analyzed per lane. As a loading control, human α-tubulin was measured. Incubation with the primary and secondary antibodies was performed overnight at 4 °C and for 1 h at room temperature, respectively. Protein bands were detected using the SuperSignal West Pico PLUS Chemiluminescent Substrate (34580, Thermo Scientific, Waltham, MA, USA). Rat monoclonal antibody against human carboxypeptidase A1 (MAB2856, R&D Systems Minneapolis, MN, USA) was used at 1:500 dilution. Mouse monoclonal antibody (DM1A) against α-tubulin (CP06, MilliporeSigma, St. Louis, MO, USA) was used at 1:2000 dilution. BiP was detected by a rabbit polyclonal anti-GRP78 antibody (ab21685, Abcam, Cambridge, MA, USA) used at 1:2000 dilution. Horse-radish peroxidase (HRP)-conjugated goat polyclonal anti-rat IgG (HAF005, R&D Systems, Minneapolis, MN, USA) was used at 1:1000 dilution. HRP-conjugated goat polyclonal anti-mouse IgG (HAF007, R&D Systems) was used at 1:2500 dilution. HRP-conjugated goat polyclonal anti-rabbit IgG (HAF008, R&D Systems) was used at 1:2000 dilution.

### 4.10. Reverse-Transcription (RT) Quantitative PCR (qPCR) to Measure HSPA5 (BiP) and DDIT3 (CHOP) mRNA

Total RNA was extracted from the transfected cells using the RNeasy Plus Mini Kit (74136, Qiagen, Valencia CA, USA). RNA (2 µg) was reverse-transcribed using the High Capacity cDNA Reverse Transcription Kit (4368814, Life Technologies). The TaqMan Gene Expression Assays and TaqMan Universal PCR Mastermix (4364338, Life Technologies) were used to determine mRNA expression levels for *HSPA5* (Hs00607129_gH) and *DDIT3* (Hs00358796_g1) with *GAPDH* (Hs02758991_g1) as the house-keeping reference gene. Expression was quantitated using the comparative cycle threshold method (ΔΔCT method). Individual CT values were first normalized to those of *GAPDH* (ΔCT) and then to the average ΔCT of the empty vector (ΔΔCT). Results were expressed as fold changes calculated with the formula 2^−ΔΔCT^.

### 4.11. Measurement of X-Box Binding Protein 1 (XBP1) mRNA Splicing

ER stress associated splicing of the *XBP1* mRNA was studied by RT-PCR using the following primers that amplified both the spliced (415 bp) and unspliced (441 bp) forms: *XBP1* sense primer, 5′-CCT TGT AGT TGA GAA CCA GG-3′; *XBP1* antisense primer, 5′-GGG CTT GGT ATA TAT GTG G-3′. *GAPDH* was amplified (352 bp) as the house-keeping control gene with the following primers: *GAPDH* sense primer, 5′-AAG GTC GGA GTC AAC GGA TTT-3′; *GAPDH* antisense primer, 5′-AGA TGA TGA CCC TTT TGG CTC-3′. The PCR amplicons were resolved on 2.5% agarose gels and stained with GreenGlo Safe DNA Dye (C788T73, Thomas Scientific, Swedesboro, NJ, USA).

## Figures and Tables

**Table 1:** Natural *CPA1* variants in the disulfide-stabilized loop sequence published to date. CP, chronic pancreatitis. Note that variant p.A259V was not included in this study.

Exon	Nucleotide Change	Amino-Acid Change	Number of CP Carriers	Number of Non-CP Carriers	Gnomad Allele Frequency
exon 7	c.749G>C	p.G250A	1	0	absent
exon 7	c.751G>A	p.V251M	7	0	6/251,446
exon 7	c.758C>G	p.P253R	1	0	absent
exon 7	c.764G>T	p.R255M	1	0	absent
exon 7	c.768C>G	p.N256K	7	0	absent
exon 7	c.775G>A	p.A259T	0	1	1/31,398
exon 7	c.776C>T	p.A259V	0	1	3/282,782
exon 8	c.809C>G	p.P270R	1	0	absent
exon 8	c.811T>C	p.C271R	2	0	absent

**Table 2:** Secretion of *CPA1* variants from transfected HEK 293T cells and CPA1 activity of the activated conditioned medium (given as % of wild-type CPA1 secretion and activity, mean ± standard deviation, *n* = 5–9). Values published previously are also listed [13].

Mutation	Secretion (%) This Study	Secretion (%) Published	Activity (%) This Study	Activity (%) Published
p.G250A	0	--	0	--
p.V251M	0	0	0	0
p.P253R	0	0	0	0
p.R255M	69.8 ± 10.9	86	0	0
p.N256K	0	0	0	0
p.A259T	87.4 ± 3.4	82	73.2 ± 13.3	85
p.P270R	14.4 ± 2.6	14	10.0 ± 6.3	9
p.C271R	0	0		1

## Data Availability

All experimental data are contained within the article.
